# InAs/InAsSb Strain-Balanced Superlattices for Longwave Infrared Detectors

**DOI:** 10.3390/s19081907

**Published:** 2019-04-22

**Authors:** Tetiana Manyk, Krystian Michalczewski, Krzysztof Murawski, Piotr Martyniuk, Jaroslaw Rutkowski

**Affiliations:** 1 Institute of Applied Physics, Military University of Technology, 2 Urbanowicza Str., 00-908 Warsaw, Poland; krystian.michalczewski@wat.edu.pl (K.M.); krzysztof.murawski01@wat.edu.pl (K.M.); piotr.martyniuk@wat.edu.pl (P.M.); jaroslaw.rutkowski@wat.edu.pl (J.R.); 2 VIGO System S.A. 129/133 Poznanska Str., 05-850 Ozarow Mazowiecki, Poland; kmichalczewski@vigo.com.pl

**Keywords:** T2SLs InAs/InAsSb, energy bandgap, infrared detectors, LWIR

## Abstract

The InAs/InAsSb type-II superlattices (T2SLs) grown on a GaSb buffer layer and GaAs substrates were theoretically investigated. Due to the stability at high operating temperatures, T2SLs could be used for detectors operating in the longwave infrared (LWIR) range for different sensors to include, e.g., CH_4_ and C_2_H_6_ detection, which is very relevant for health condition monitoring. The theoretical calculations were carried out by the 8 × 8 k·p method. The estimated electrons and heavy holes probability distribution in a InAs/InAsSb superlattice (SL) shows that the wave function overlap increases while the thickness of the SL period decreases. The change in the effective masses for electrons and holes versus the SL period thickness for the k_z_-direction of the Brillouin zone is shown. The structures with a period lower than 15 nm are more optimal for the construction of LWIR detectors based on InAs/InAsSb SLs. The experimental results of InAs/InAsSb T2SLs energy bandgap were found to be comparable with the theoretical one. The proper fitting of theoretically calculated and experimentally measured spectral response characteristics in terms of a strain-balanced and unbalanced structures is shown.

## 1. Introduction

Smith et al. [1] in 1987 proposed the use of type-II superlattices (T2SLs) InAs/GaSb as optoelectronic materials exhibiting excellent electro-optical properties, theoretically comparable to HgCdTe being the main compound for detection in the infrared radiation (IR) region [2]. In addition, Ga-free T2SLs InAs/InAs_1−x_Sb_x_ have been proved to have a longer carrier lifetime (*τ*) than InAs/GaSb T2SLs [3] and have been proposed as an alternative for IR photodetectors [4]. The molecular beam epitaxy (MBE) technology development allowed investigation of the superlattices (SLs) InAs/InAsSb on a GaSb buffer layer grown on GaAs substrates. The InAs_1−x_Sb_x_ energy bandgap (*E_g_*) changes non-linearly versus the Sb molar composition in the ternary compound. The form of this relation depends on the InAs_1−x_Sb_x_ growth method and is given in different forms by research groups [5,6].

In the InAs/InAsSb SLs due to the T2SLs band alignment the electron and hole states are confined within the InAs (electrons) and InAsSb (holes) layers, and thus electrons and holes are spatially separated. Therefore, by adjustment of the InAs and/or InAsSb thickness as well as the Sb molar composition (*x_Sb_*), it is feasible to tune *E_g_* within a wide range of IR. Owing to these unique properties, T2SLs InAs/InAsSb have been chosen as materials for applications in the longwave infrared radiation (LWIR) detectors operating within the 8–15 μm range. Both photoconductive and photovoltaic detectors are constructed. T2SLs InAs/InAsSb are mainly used for the fabrication of barrier detectors to include nBn and pBn design [7,8], low-noise interband cascade infrared photodetectors (ICIP) [9,10], dual band long-wavelength infrared photodiodes [11] and very fast avalanche photodiodes (APD). These detectors can be used for a wide range of applications in the fields of science, medicine, safety, industry, and automotive, such as railway safety, gas leak detection, flame detection, heat distribution monitoring, medical diagnostic imaging, space operations, night vision devices and spectroscopy. Since humanity is facing healthcare challenges that include the rising and potentially unsustainable health and care costs, mainly due to the increasing prevalence of chronic diseases, and the influence on health of external environmental factors including climate change, there is a need to have simple devices allowing us to detect and diagnose e.g., lipid peroxidation, vitamin E deficiency, chronic respiratory disease, cells oxidative stress, or even scleroderma and cystic fibrosis. This could be allowed by e.g., the C_2_H_6_ level monitoring through unexplored LWIR, requiring a proper detector operating in that range. In addition, in the LWIR, the two main detrimental scattering effects of Rayleigh and Mie are significantly reduced [12,13]. 

The T2SL system was modeled using a variety of theoretical approaches, such as the tight-binding, pseudopotential and k∙p methods. The k∙p method is widely used because of its proper numerical accuracy. We present a comparison of the responsivity of high operating temperature (HOT) LWIR InAs/InAs_1−x_Sb_x_ T2SLs photoconductors with simulated absorption spectra. 

## 2. Materials and Methods

**Figure 1:**
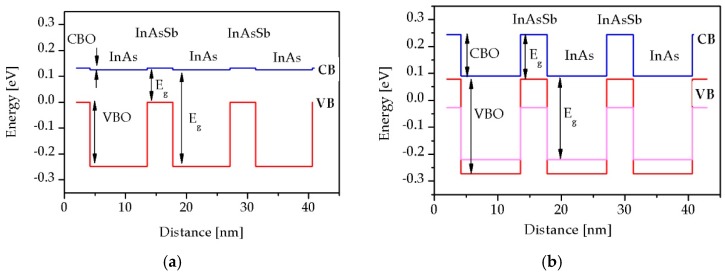
Schematic representation of (**a**) unstrained and (**b**) strained type-II superlattices (T2SLs) InAs/InAs_1−x_Sb_x_ SLs.

The theoretical calculation of band structure, *E_g_* and absorption coefficient (α) was performed using the standard k∙p (8 × 8 method) by the SimuApsys and nextnano platforms [14,15]. In Figure 1, the schematic representation of the energy bands of the strained and unstrained InAs/InAsSb SL is presented. 

Figure 1 shows that when the SL is coherently strained, all energy parameters are being changed to include conduction band offset (CBO), valence band offset (VBO) and *E_g_* of the InAs (InAsSb) layers. The blue line corresponds to the lowest conduction band (CB), and the red line indicates the highest valence band (VB). If the SL lattice-match to the GaSb substrate is taken into account, the InAs and InAs_1−x_Sb_x_ layers become strained and valence band splits to the heavy (hh) and light (lh) hole sub-bands in InAs/InAsSb SLs. In Figure 1 the violet line represents the lh subband edge in the strained SLs and the separation of the hh and lh bands is also visible. The change in VBO value between the state without strain and strained remains within 0.1 eV [5].

The k∙p (8 × 8 method) simulation was implemented to study dispersion curves in order to estimate the SLs’ effective masses. The periodic boundary conditions were used in the simulation procedure. These conditions impose periodicity on the wave functions (WF). 

The parameters of InAs, InSb and GaSb layers used in InAs/InAs_1−x_Sb_x_ SLs simulation procedure are presented in Table 1. Parameters are taken from the papers of several authors [5,16,17,18,19,20,21]. *E_g_* versus temperature (*T*) is given by the Varshni form: $E_{g}\left(T\right){=E}_{0}{-\alpha T}^{2}/\left(T+\beta \right)$.

All parameters for ternary compounds used for simulations were estimated based on the bulk parameters for binary compounds. It should be noted that we considered the symmetrical case when the Kane parameter, *F* = 0 [15]. Most parameters assumed in simulations exhibit *T* dependence and vary linearly with *x_Sb_*. In order to calculate some InAs_1−x_Sb_x_ (0 < *x_Sb_* < 1) parameters the bowing shown in the Table 2 was used.

An equation for ternary compounds parameters, Y_InAsSb_ dependence on the bowing coefficient is defined as:

(1) $$Y_{\mathrm{InAsSb}}=(1-x_{\mathrm{Sb}})\times Y_{\mathrm{InAs}}+x_{\mathrm{Sb}}\times Y_{\mathrm{InSb}}-b_{\mathrm{bow}}\times x_{\mathrm{Sb}}\times (1-x_{\mathrm{Sb}}).$$

The Luttinger parameters have been estimated based on respective effective masses according to equations presented by Birner et al. [15] and Vurgaftman et al. [16]. The VBO was determined by the following equation:

(2) $${\mathrm{VBO}=E}_{v,\mathrm{vac}(\mathrm{InAs})}-\left[E_{v,\mathrm{vac}(\mathrm{InAs})}{\times (1-x_{\mathrm{Sb}})+E}_{v,\mathrm{vac}(\mathrm{InSb})}\times (x_{\mathrm{Sb}})-x_{\mathrm{Sb}}\times b_{v,\mathrm{vac}}\times (1-x_{\mathrm{Sb}})\right],$$

where the vacuum energy levels of the valence band (E_v_, _vac_) and the valence band bowing parameter (b_v_, _vac_) were presented in Table 1 and Table 2, respectively.

All calculated SL structures are strain-balanced on GaSb. The strain-balanced condition is reached by setting the average lattice parameter of one period weighted with the layer thickness being equal to the lattice constant of GaSb. The InAs_1−x_Sb_x_ layer thickness (d_InAsSb_) versus *x_Sb_*, lattice constant (a_InAs(InSb, GaSb)_) and SL period (*L*) can be calculated from Equation [22]:

(3) $$d_{\mathrm{InAsSb}}=\left(\frac{a_{\mathrm{GaSb}}{-a}_{\mathrm{InAs}}}{a_{\mathrm{InSb}}{-a}_{\mathrm{InAs}}}\right)\times \frac{L}{x_{\mathrm{Sb}}}.$$

The lattice constant at 300 K is presented in Table 1. The thickness of the InAsSb layer in InAs/InAsSb SL structures was determined by relation (3) giving the thickness of the layer being compensated to zero sum of the strains of all constituent SL layers.

## 3. Results and Discussion

### 3.1. Influence of the Period on the Superlattice (SL) Parameters

**Figure 2:**
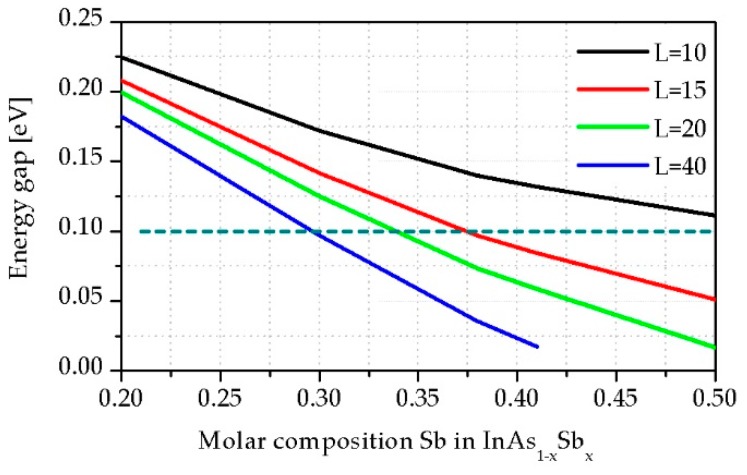
Theoretically simulated *E_g_* for the InAs/InAsSb strain balanced superlattice (SL) structures versus *x_Sb_* at *T* = 230 K.

Figure 2 shows the lowest transition energy (*E_g_*-between the first level of CB e_1_ and VB hh_1_) versus *x_Sb_* in InAsSb for SL periods between 10 nm and 40 nm at *T* = 230 K. 

Figure 2 depicts that it is possible to reach the desired *E_g_* by changes in both *x_Sb_* and *L*. When *x_Sb_* and *L* increase, *E_g_* decreases.

**Figure 3:**
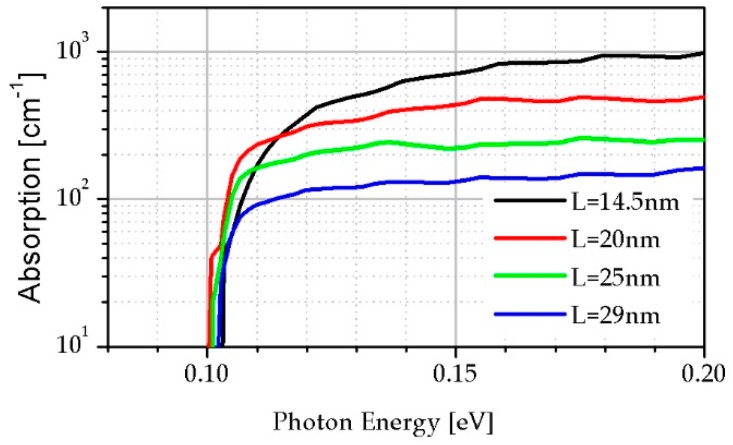
*α* theoretical simulation versus photon energy for the strain-balanced SL InAs/InAsSb.

The broken line in Figure 2 indicates an energy equal of 0.1 eV, which allows us to compare the difference between the thickness for the InAs/InAsSb strain-balanced SL structures with the same *E_g_*. The absorption coefficient presented in Figure 3 was calculated by the commercial SimuApsys platform according to the method presented in [23] for selected points from this line with another SL period. 

Figure 3 shows that *α* near the edge of absorption decreases when thickness of the InAs/InAsSb SL increases, being connected with the overlap of the electrons and holes WF in InAs/InAsSb SL. 

**Figure 4:**
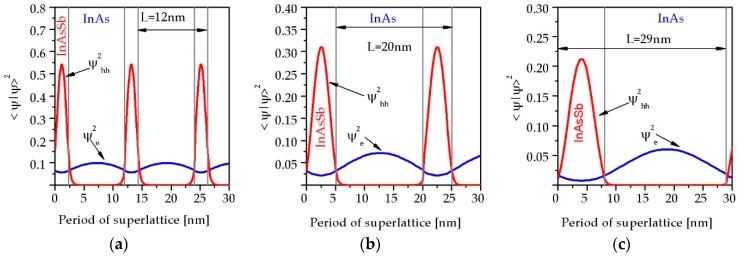
Electrons and heavy holes probability distribution for InAs/InAsSb SL structures at 230 K for the SL period: (**a**) 12 nm; (**b**) 20 nm; (**c**) 29 nm.

The probability distribution for electrons and heavy holes in InAsSb/InAs SLs is presented in Figure 4. These simulations are at *T* = 230 K for *x_Sb_* = 0.38 and SL period *L* = 12 nm, 20 nm and 29 nm, respectively. The heavy hole WF is strongly localized within the InAsSb barrier region but electron WF spreads out through the structure with significant probability of residing in the InAs quantum well (QW) region. Figure 4 shows that if the period of the InAs/InAsSb strain-balanced SL increases the overlap of the electrons and holes, WF decreases. The values of the overlap are 21.4%, 14.8%, 8.7% for the SL period *L* = 12 nm, 20 nm, 29 nm, respectively. The decrease in overlap confirms the reduction of *α* when SL period increases.

**Figure 5:**
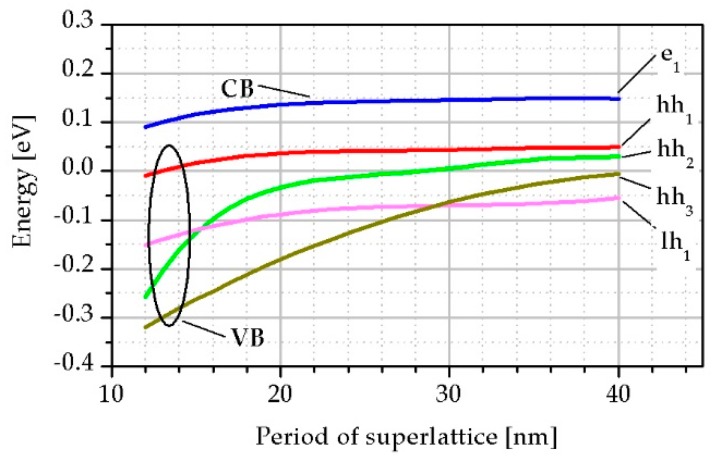
The CB e_1_ and VB: hh_1_, hh_2_, hh_3_, lh_1_ positions versus SL period.

Thin period InAs/InAsSb SL structures shows that better optical properties and thinner layers in SL are more stable in terms of the electrons and holes interaction in deeper energy levels. As can be seen from Figure 5, when the period of SL is above 15 nm, the position of lh and hh sub-bands is changed affecting on *α*. In SL with a period exceeding 30 nm three bands of heavy holes: hh_1_, hh_2_ and hh_3_ lies above the light hole sub-band lh_1_. 

**Figure 6:**
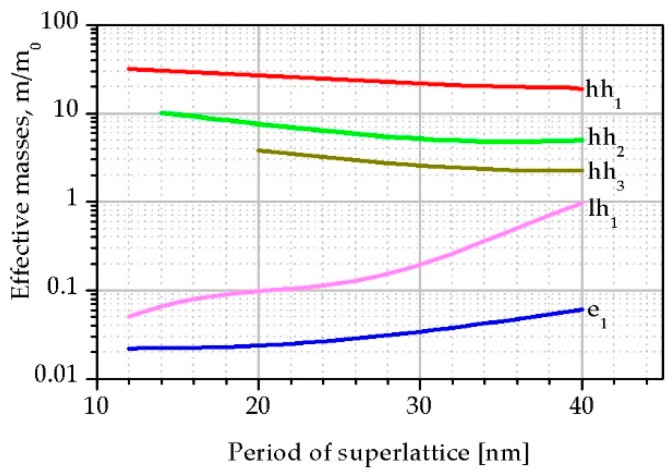
The calculated e_1_ and hh_1_, hh_2_, hh_3_ and lh_1_ effective masses for the k_z_-direction of the Brillouin zone versus SL period.

The electrons and holes effective masses were calculated from the dispersion curves as the second derivative of energy by the wave vector (k). For directions in the k_x_, k_y_ of the Brillouin zone effective masses hardly change with the SL period but for direction k_z_ dependence of the effective masses versus SL period is significant (see Figure 6). When *x_Sb_* increases the hh effective masses decreases while both lh and e_1_ masses increase. It can be seen that an increase in the InAs/InAsSb SL period leads to more significant change in the lh_1_ mass. Moreover, it should be noted that with a change in the SL period in the range of 10–20 nm, the lh mass slowly increases and stays constant to 0.1m_0_ while with a further increase in SL period those masses increase and become similar to the hh masses. 

In the case of T2SLs InAs/InAs_0.62_Sb_0.38_ with the *L* = 14.5 nm at *T* = 230 K, the calculated effective masses of e_1_, hh and lh for directions in the k_x_, k_y_ (||) and k_z_ (⊥) of the Brillouin zone are: $m_{e||}^{*}$ = 0.019m_0_, $m_{e⊥}^{*}$ = 0.023m_0_, $m_{\mathrm{hh}||}^{*}$ = 0.040m_0_, $m_{\mathrm{hh}⊥}^{*}$ = 31.02m_0_, $m_{\mathrm{lh}||}^{*}$ = 0.104m_0_, $m_{\mathrm{lh}⊥}^{*}$ = 0.096m_0_. 

**Figure 7:**
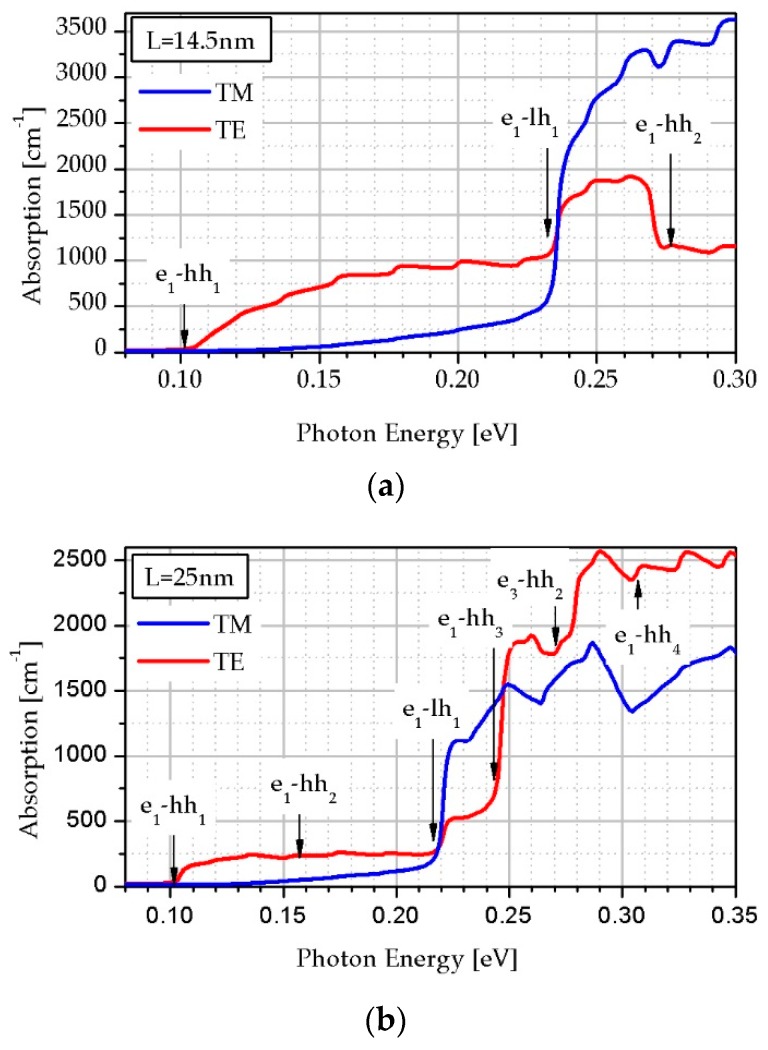
The polarization-dependent transverse electronic (TE) and transverse magnetic (TM) *α* versus energy in InAs/InAs_0.62_Sb_0.32_ SL structures at 230 K for the SL period equal to: (**a**) 14.5 nm, (**b**) 25 nm.

In order to explain the shape of the absorption curves in a wider photon energy range, it is necessary to consider the polarization of the light [24]. Figure 7 shows *α* for both transverse electronic (TE) and transverse magnetic (TM) mode in the LWIR region for two different SL periods equal to *L* = 14.5 nm and *L* = 25 nm. These theoretical calculations show four prominent transition channels, namely e_1_-hh_1_, e_1_-hh_2_, e_1_-hh_3_ and e_1_-lh_1_ contributing to the TE and TM absorption in this wavelength region. The absorption strength for TE is higher than that for TM polarization.

**Figure 8:**
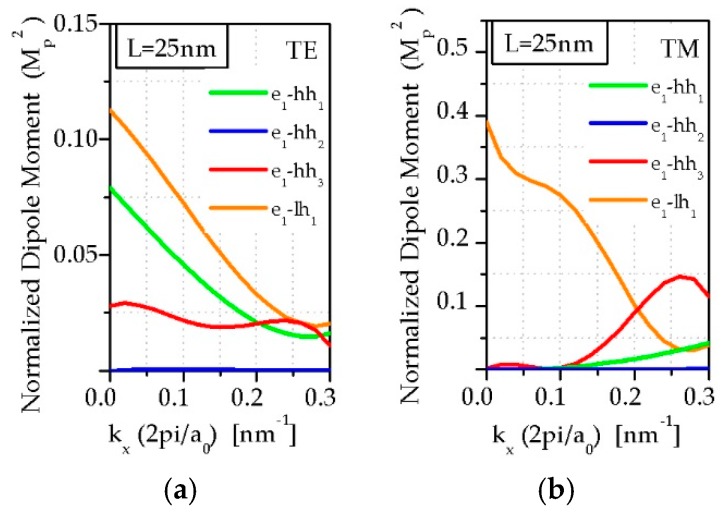
(**a**) TE and (**b**) TM—polarized the optical matrix for the InAs/InAs_0.62_Sb_0.32_ strain balanced SL for *L* = 25 nm at *T* = 230 K.

The transition probability is proportional to the square of the optical matrix element per period. Figure 8 presents the optical matrix elements for the e_1_-hh_1_, e_1_-hh_2_, e_1_-hh_3_ and e_1_-lh_1_ transition for the TE and TM polarization for the InAs/InAs_0.62_Sb_0.32_ strain-balanced SL with the *L* = 25 nm at *T* = 230 K. The optical matrix element for the e_1_-hh_1_ transition is larger for TE polarization than for TM. In turn, the TE-polarized absorption increases sharply near *E_g_* due to the e_1_-hh_1_ transition, while the TM absorption increases markedly at higher energies due to e_1_-lh_1_ transition. Figure 8 shows that the optical matrix element e_1_-hh_2_ has no effect on the coefficient *α*, since its value is almost zero. 

Typical spectra contains two main features where one is an increase of absorption (e_1_-hh_1_) while the second one is a peak at higher energy corresponding to the transition between other bands. From the theoretical descriptions presented above, it follows that two transitions have a great influence on *α*, namely e_1_-hh_1_ and e_1_-lh_1_ (see Figure 7).

### 3.2. Comparison of the Theoretical Simulation with the Experimental Data

The T2SLs InAs/InAsSb were grown on GaAs substrates by a RIBER Compact 21-DZ solid-source MBE system. A 1.2 µm-thick GaSb layer was grown to reduce the large lattice mismatch between GaAs substrate and the SLs. Then, 300 periods 34.3 ML InAs/9 ML InAsSb SLs were deposited. Growth details of InAs/InAsSb SL are reported in the paper by Michalczewski et al. [25]. After the growth, photolithography-assisted wet-etching was used to define the active and contact areas of the IR photoconductors. The vacuum evaporation of Au/Ti was applied to fabricate ohmic contacts. 

**Figure 9:**
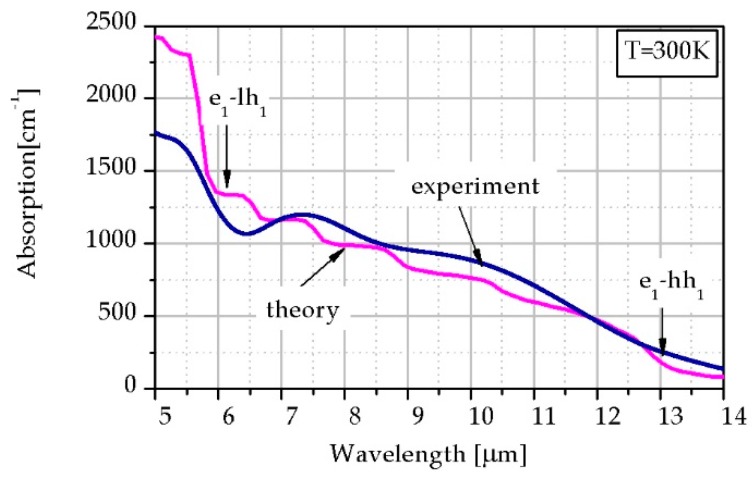
*α* theoretical simulation and experimental data for T2SLs InAs/InAs_0.62_Sb_0.38_ with *L* = 13.2 nm at *T* = 300 K.

In order to obtain a proper agreement between theoretical calculations and experimental data, the temperature-dependent bowing parameter for *E_g_* was used. In our simulation the bowing parameter decreases versus *T* assuming 0.67 meV at 300 K. Figure 9 shows the theoretical simulation and experimental data of *α* for 1.7 μm thick T2SLs InAs/InAs_0.62_Sb_0.38_ with *L* = 13.2 nm SL at 300 K. We have reached proper agreement between the experimental data and theoretical simulation. Figure 9 shows the transition e_1_-hh_1_ being equal to *E_g_* and the second one corresponds to e_1_-lh_1_ transition.

**Figure 10:**
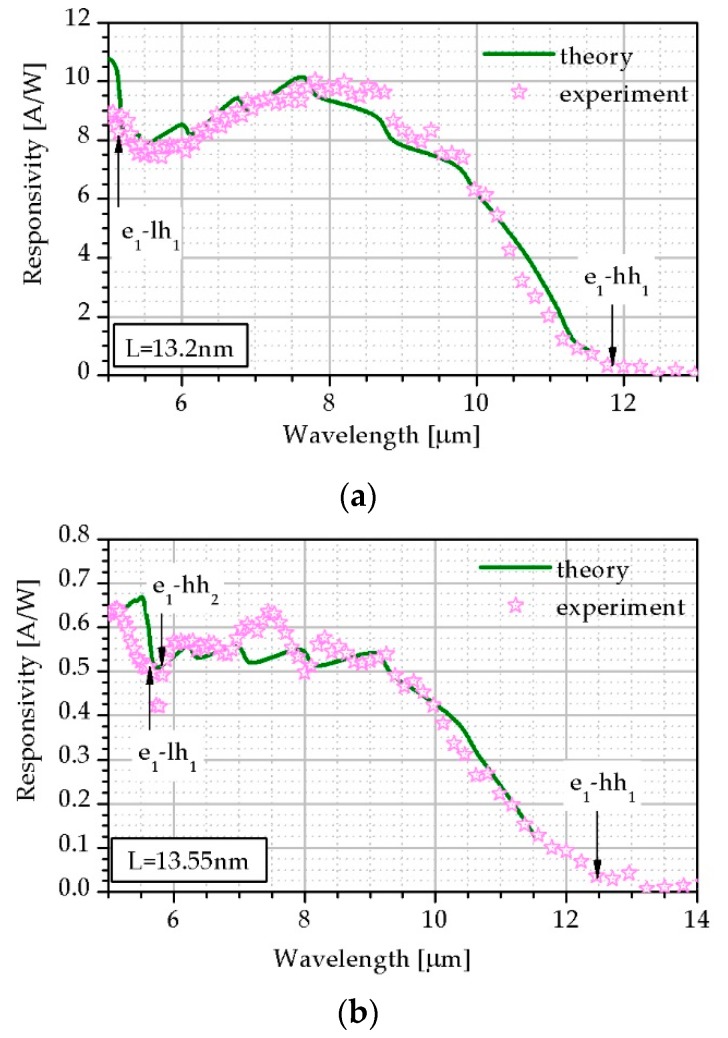
*R_i_* theoretical simulation and experimental results for T2SLs InAs/InAs_0.62_Sb_0.38_ photoconductor for (**a**) sample-A; (**b**) sample-B at *T* = 230 K.

T2SLs photoconductor spectral current responsivity (*R_i_*) operating in LWIR range at bias 0.5 V and *T* = 230 K was measured and results are presented in Figure 10 where green line depicts theoretical fitting while pink stars correspond to the experimental results for two samples, A and B. The SL period, the thickness InAs and InAs_1−x_Sb_x_, *x_Sb_*, *E_g_* and the fitting *τ* are presented in Table 3. 

In the case of theoretical calculations the *R_i_* was estimated by *α* and *τ* fitting. Theoretical calculations exhibit proper coincidence with experimental data. 

Figure 10 shows a comparison of *R_i_* theoretical simulation and experimental results for the two samples. Their common feature is the same composition (*x_Sb_* = 0.38) while the difference is the thickness of the SLs (the ratio of the thicknesses InAs and InAsSb) and *E_g_*. Figure 10a corresponds to sample-A where thickness is located in the strain-balanced region while Figure 10b depicts sample-B exhibiting unbalanced strain structure. The fitting to experimental results was performed by *τ.* Table 3 indicates that *τ* for sample-B is much lower than for sample-A explaining the difference in *R_i_*. We believe that the structure by which the responsivity is presented in Figure 10a is optimally balanced. Thus, the optimal structure of T2SLs InAs/InAsSb for LWIR photodetectors should have an strain-balanced absorber with the following parameters: SL period of 12 nm < L < 15 nm and Sb molar composition in InAs_1−x_Sb_x_ of 0.37 < x < 0.45.

## 4. Conclusions

The T2SLs InAs/InAs_1−x_Sb_x_ SLs on a GaSb buffer and GaAs substrate were investigated. The use of InAs/InAs_1−x_Sb_x_ SLs gives a great opportunity to fabricate devices for IR radiation detection in a wide wave range by selecting the SL period and the Sb molar composition of the InAs_1−x_Sb_x_ barrier. In the calculations, the thickness of the InAsSb barrier for the given period was assumed to ensure a strain balanced SLs. The paper shows that the reduction in period thickness and increase in Sb molar composition in the InAsSb barrier allows higher absorption to be reached for the same absorption edge. An absorption improvement is caused by the increase of the electrons and holes wave function overlap due to the thinner and lower InAsSb barrier. With the change in the thickness of the SL period, the position of the light and heavy hole bands changes significantly. With a period smaller than 15 nm, the light holes band is located directly under the heavy holes band, while for periods larger than 30 nm it is separated from the conduction band by three heavy holes bands. Numerical simulations show that the position of the light holes sub-band directly below the first heavy holes sub-band increases the probability of optical transitions, hence structures with a smaller period are more optimal for the construction of LWIR detectors based on InAs/InAsSb SLs. 

The experimental data were compared with the results of numerical simulations, showing proper agreement by lifetime fitting. The estimated carrier lifetime for a balanced structure is more than an order of magnitude higher than for a non-balanced one indicating that the generation of a large number of defects in the unbalanced structure increases the recombination rate of carriers reducing the carrier lifetime and current responsivity. 

## Figures and Tables

**Table 1:** Material parameters of InAs, InSb and GaSb layers used in simulation.

Parameter	InAs	InSb	GaSb
E_0_(Γ) [eV]	0.417	0.235	0.812
E_0_(X) [eV]	1.433	0.63	1.141
E_0_(L) [eV]	1.133	0.93	0.875
∆_so_ [eV]	0.39	0.81	0.76
m_e_/m_0_ (0K)	0.026	0.014	0.039
E_v_, _vac_	1.39	1.75	1.78
a = f (T) [Å]	$6.0583+2.47{\times 10}^{-5}\left(T-300\right)$	$6.4794+3.48{\times 10}^{-5}\left(T-300\right)$	$6.0959+4.72{\times 10}^{-5}\left(T-300\right)$

**Table 2:** Bowing parameter assumed in simulations (*T* = 230 K).

Bowing Coefficient	Bowing Parameter (b_bow_)
b_g_ [eV]	0.72
b_∆so_ [eV]	1.2
b_me/m0_	0.035
b_v_, _vac_ [eV]	– 0.47

**Table 3:** The sample-A and sample-B parameters.

Sample	*L* [nm]	*L*_InAsSb_ [nm]	*L*_InAs_ [nm]	*x_Sb_*	*E_g_* [eV]	*τ* [ns]
A	13.20	2.8	10.4	0.38	0.1048	24
B	13.55	4.2	9.35	0.38	0.0996	1.2
